# Biocontrol Potential of *Streptomyces odonnellii* SZF-179 toward *Alternaria alternata* to Control Pear Black Spot Disease

**DOI:** 10.3390/ijms242417515

**Published:** 2023-12-15

**Authors:** Fei Zhang, Shaohua Wen, Beibei Wang, Zhe Zhang, Fang Liu, Ting Ye, Kaimei Wang, Hongju Hu, Xiaoping Yang, Wei Fang

**Affiliations:** 1 National Biopesticide Engineering Research Centre, Hubei Biopesticide Engineering Research Centre, Hubei Academy of Agricultural Sciences, Wuhan 430064, China; feifeisjtu@163.com (F.Z.); wenshaohua1985@163.com (S.W.);; 2 Hubei Hongshan Laboratory, Wuhan 430070, China; 3 Hubei Key Laboratory of Germplasm Innovation and Utilization of Fruit Trees, Research Institute of Fruit and Tea, Hubei Academy of Agricultural Science, Wuhan 430064, China

**Keywords:** *Streptomyces odonnellii* SZF-179, *Alternaria alternata*, pear black spot, biocontrol, fermentation broth

## Abstract

Pear black spot disease, caused by *Alternaria alternata,* is a devastating disease in pears and leads to enormous economic losses worldwide. In this investigation, we isolated a *Streptomyces odonnellii SZF-179* from the rhizosphere soil of pear plants in China. Indoor confrontation experiments results showed that both SZF-179 and its aseptic filtrate had excellent inhibitory effects against *A. alternata*. Afterwards, the main antifungal compound of SZF-179 was identified as polyene, with thermal and pH stability in the environment. A microscopic examination of *A. alternata* mycelium showed severe morphological abnormalities caused by SZF-179. Protective studies showed that SZF-179 fermentation broth could significantly reduce the diameter of the necrotic lesions on pear leaves by 42.25%. Furthermore, the potential of fermentation broth as a foliar treatment to control black leaf spot was also evaluated. Disease indexes of ‘Hosui’ and ‘Wonwhang’ pear plants treated with SZF-179 fermentation broth were lower than that of control plants. Overall, SZF-179 is expected to be developed into a safe and broad-spectrum biocontrol agent. No studies to date have evaluated the utility of *S. odonnellii* for the control of pear black spot disease; our study fills this research gap. Collectively, our findings provide new insights that will aid the control of pear black spot disease, as well as future studies of *S. odonnellii* strains.

## 1. Introduction

The pear (*Pyrus* spp.) is one of the most consumed fruits in view of its high nutritional and economic relevance worldwide. However, they are highly susceptible to pathogenic fungal infections during the growing season, harvest and storage. *Alternaria alternata* (*A. alternata*), the pathogen in black spot disease, has been widely reported in pear. This disease first appears as brown necrotic lesions on fruits or leaves, which may further merge into larger necrotic areas, resulting in leaf drop or fruit tissue decay pre-harvest [1,2,3]. During storage, *A. alternata* not only causes serious rotting in fruit tissues, but also causes the accumulation of mycotoxins that lower pear quality, shorten the storage period and reduce market value [4,5], ultimately leading to serious economic losses and food safety problems [6]. Owing to its wide existence in nature in the form of saprophytes or pathogens, a broad host range and the significant transmission ability and pathogenicity, *A. alternata* is difficult to prevent and control [7,8]. Therefore, effective control of pear black spot disease has become a crucial issue for the development of the pear industry.

Currently, chemical fungicides such as azoxystrobin, prochloraz [9], tebuconazole [10] and difenoconazole [11] are commonly used to prevent and control the pear black spot disease. However, the frequent use of a high dose of these fungicides has caused the emergence of fungicide-resistant strains and environmental contamination [12]. Public awareness of food safety has increased and led to widespread acknowledgement of the necessity of safe and effective plant disease control methods. Concomitantly, biocontrol approaches have increasingly been applied as an alternative to traditional chemical methods for controlling various plant diseases. Furthermore, in recent years, biocontrol organisms have exhibited a good control potential in the prevention and control of pear diseases. Inoculation of a Portuguese isolate of *Aureobasidium pullulans* at 3 × 10^8^ and 4 × 10^9^ CFU/mL reduced the incidence of the blue mold disease in “Rocha” pears by 23% and 63%, and reduced the lesion diameter by 36% and 46%, respectively [13]. Cold stored *Vishniacozyma victoriae* NPCC 1263for 60 days and 15 days could reduce the incidence of postharvest fungal diseases in organic pears by 71% and 92%, respectively [14].

Among several groups of antagonistic microorganisms, *Actinomycetes*, a group of Gram-positive (G^+^) filamentous bacteria, are widely known for their production of secondary metabolites which are antibacterial, antifungal, antiviral, antiprotozoal, antihelminth, anticancer, anticholesterol and immunosuppressant [15]. *Streptomyces*, a genus belonging to the order *Actinomycetales*, are widely documented for their broad metabolic capabilities and their ability to produce antibiotics, pigments and hydrolytic enzymes, making them a fascinating group for biocontrol applications. Of the commercialized antibiotics, such as the insecticides avermectin [16], the fungicide Jinggangmycin [17] and the antiviral Ningnanmycin [18,19], 90% are produced by *Streptomyces*. More biocontrol products from *Streptomyces* are already being marketed and the discovery of natural products is still ongoing [20,21]. So, as a promising resource of biocontrol agents, *Streptomyces* holds enormous research and development potential.

*Streptomyces odonnellii* (*S. odonnellii*) was first isolated from a soil sample collected from the Brazilian Cerrado (savanna) in 2001 [22]. Only the description of its morphological and physiological characterization, proteolytic activity and thermostability and chitinolytic activity of enzymes was documented; no study has examined the application of *S. odonnellii* in the control of plant diseases, especially pear black spot disease. Therefore, the objectives of this study were to evaluate the efficiency of *S. odonnellii* strain SZF-179 in the control of pear black spot disease. Here, we isolated SZF-179 from the rhizosphere soil in a pear orchard in Wuhan, China. We studied the antifungal spectrum of SZF-179 and preliminarily identified its active antifungal substance against *A. alternata*. The biocontrol potential of SZF-179 was further evaluated with respect to protective assessment and field experiment. As far as we know, this is the first report on the biocontrol ability of *S. odonnellii* in the management of pear disease. Our study provides a novel, efficient *Streptomyces* for biocontrol uses and a theoretical basis for the development of environmental-friendly biocontrol agents against pear black spot disease.

## 2. Results

### 2.1. Identification of the Fungal Pathogen

**Figure 1:**
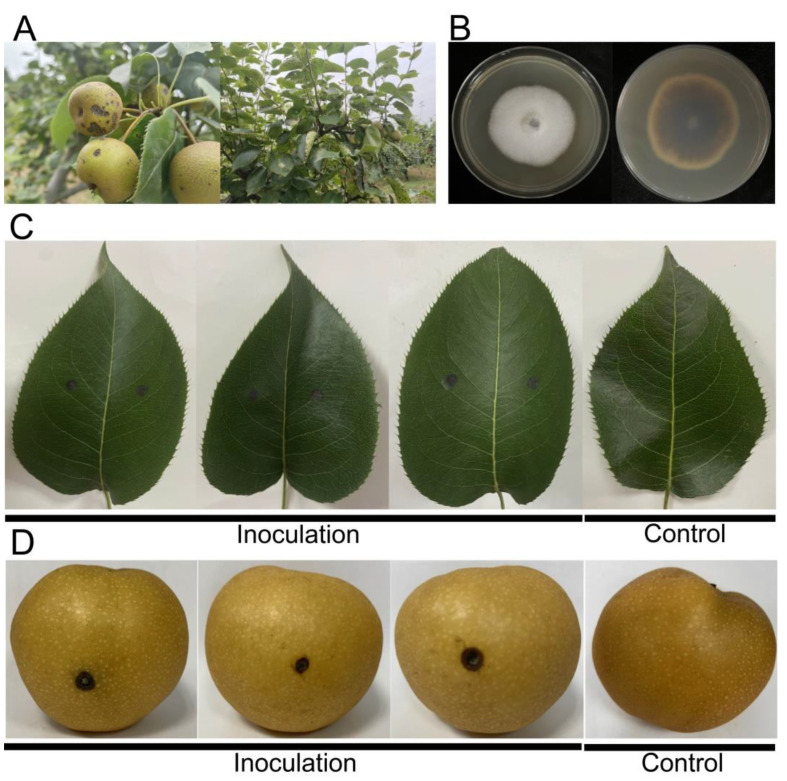
Symptoms on pear leaves and fruits, as well as morphological characteristics of *A. alternata*. (**A**) Symptom of pear leaves and fruits infected by *A. alternata* in Wuhan, China; (**B**) colony morphology of *A. alternata* on PDA cultured 3 days at 25 °C; (**C**,**D**) Re-inoculation of *A. alternata* in wounded leaves and fruits.

Field symptoms of brown-black necrotic lesions in the pear leaves and fruits were seen (Figure 1A). After being cultured on PDA for 3 days, white colonies with black pigmentation formed around the symptomatic leaves (Figure 1B), which were then purified for further identification. Genomic DNA was extracted from the isolated strain. After the universal primers ITS1 and ITS4 were used for amplification, the fungal pathogen was preliminarily identified as *A. alternata*. Then, the strain was used for re-inoculation using wounded pear leaves and fruits. The typical symptoms of black necrotic lesions, similar to those observed in the field, appeared at the inoculation sites (Figure 1C,D). *A. alternata* was re-isolated and identified using ITS sequencing, fulfilling Koch’s postulates [23].

### 2.2. Screening and Identification of Rhizosphere Bacteria against A. alternata

**Figure 2:**
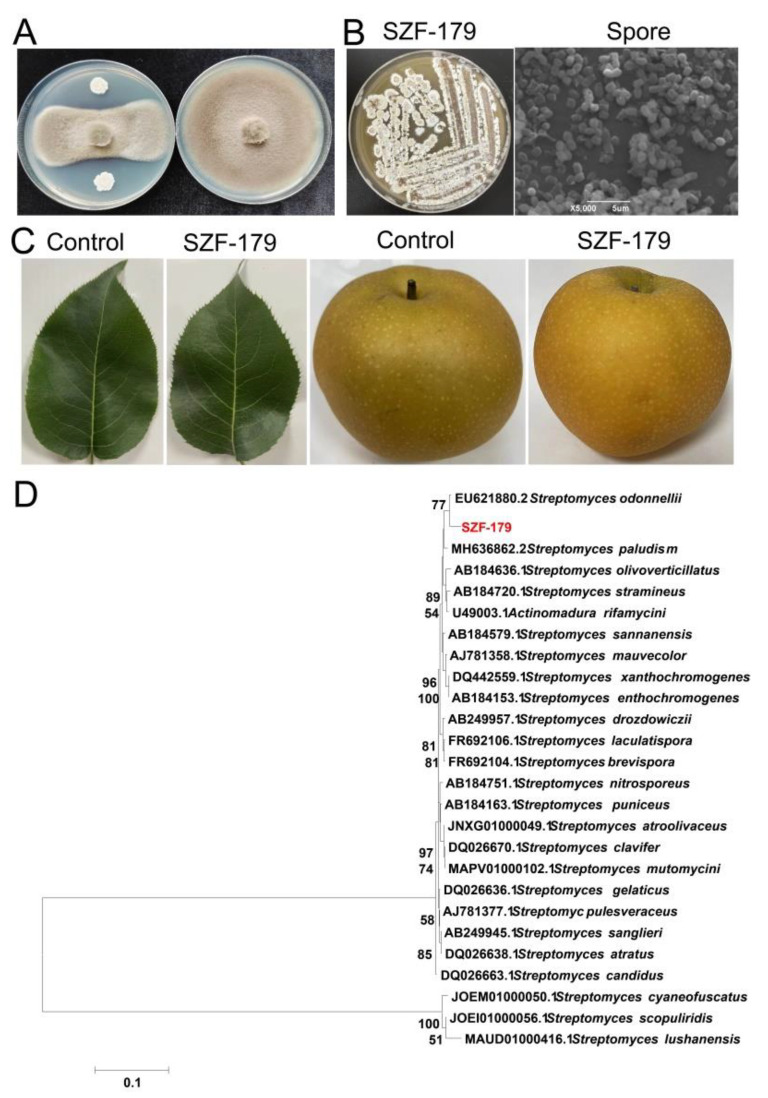
Antifungal activity of SZF-179 against *A. alternata* and morphology, phylogenetic analysis and safety of SZF-179: (**A**) Antifungal activity of SZF-179 against *A. alternata*; (**B**) colony morphology and SEM image of SZF-179 on ISP2 plate, bar represents 5 µm; (**C**) control: pear leaves and fruits inoculated with blank NA medium; SZF-179: pear leaves and fruits inoculated with SZF-179 block. (**D**) phylogenetic analysis of SZF-179 and other closely related strains based on the 16S rDNA sequence. The number at each node represents the percentage of the number of times the group of strains in that branch occurred based on 1000 bootstrap replicates.

Eight bacteria were isolated from the rhizosphere soil of pear trees with the supplement of cycloheximide and nalidixic acid. Among the isolated strains, SZF-179 showed the highest antifungal activity against the pear black spot pathogen *A. alternata* (Figure 2A). The morphological characteristics of SZF-179 colonies were milky white color and surface wrinkles with white mycelia when cultured on ISP2 plate at 28 °C for 6 days. Aerial mycelium is white and becomes grey as the spore mass is produced. Substrate mycelium is yellow-brown. Scanning electron microscopy (SEM) revealed that cells had circular spores, and the sizes of the spores were 1.6–1.8 μm × 1.7–1.8 μm (Figure 2B).

16S rRNA sequence analysis showed that SZF-179 shared 99.25% and 99.63% identities with *S. odonnellii* (EU621880.2) and *Streptomyces lushanensis* (MF077013.1), respectively. However, a neighbor-joining tree showed that SZF-179 formed a subclade with the nearest neighbor, *S. odonnellii*. So, SZF-179 was classified as *S. odonnellii* and the SZF-179 16S rRNA sequence was submitted to GenBank (accession number: OR519907). 

Blocks of SZF-179 on ISP2 plates activated for 5 days were placed on pear leaves and fruits for 3 days, and the inoculation did not cause any lesions, indicating that SZF-179 was not involved in causing any disease symptoms (Figure 2C), which also meant that the application of SZF-179 on pears was safe. 

### 2.3. Inhibitory Activity of SZF-179 against Plant Pathogens

**Figure 3:**
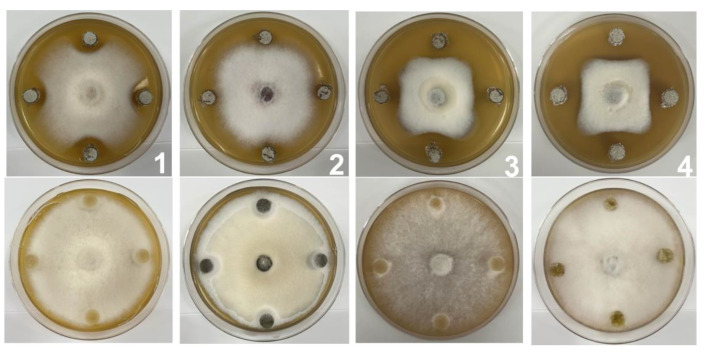
Antagonistic activity of SZF-179 against *C. gloeosporioides* (**1**), *FOL* (**2**), *P. aphanidermatum* (**3**) and *A. alternata* (**4**). Note: The top row of each group represents the treatment group, and the bottom row represents the control group.

To evaluate the biocontrol potential of SZF-179 against plant pathogens, we selected *A. alternata* and three other fungal and three bacterial pathogens for the further study. Compared with the control group, SZF-179 showed inhibitory activity against all four studied fungal pathogens: *Colletrichum gloeosporioides* (*C. gloeosporioides*), *Fusarium oxysporum* f. sp. *lycopersici* (*FOL*), *Pythium aphanidermatum* (*P. aphanidermatum*) and *A. alternata* (Figure 3), with the highest inhibitory rate (55.04%) against *A. alternata*, the lowest inhibitory rate (36.62%) against *C. gloeosporiodes*, and a moderate inhibitory rate (50.97% and 42.62%) against *P. aphanidermatum* and *FOL*, respectively (Table 1). However, SZF-179 had no inhibitory effect on the three types of bacterial pathogens, *Staphylococcus aureus* and *Micrococcus luteus* (Gram-positive, G^+^), *Escherichia coli* DH10B (Gram-negative, G^−^).

### 2.4. Effect of SZF-179 Aseptic Filtrate (AF) on Mycelial Growth of A. alternata

**Figure 4:**
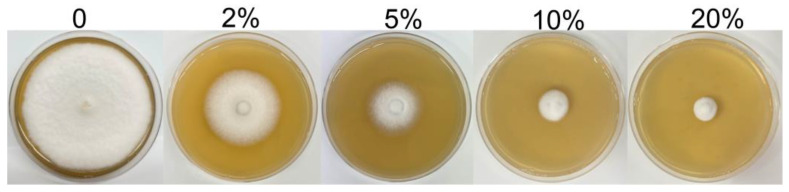
Inhibitory effect of SZF-179 AF on *A. alternata*.

To continue to confirm that SZF-179 can secrete antifungal metabolites, the inhibitory effect of SZF-179 AF on *A. alternata* mycelial growth was examined. Mycelial growth of *A. alternata* on PDA plate treated with different concentration of SZF-179 AF was determined by measuring the diameter of the *A. alternata* colony. As shown in Figure 4, the designed concentrations of SZF-179 AF could all significantly affect the growth of tested *A. alternata* and the inhibitory effect increased with increasing concentrations of the AF. SZF-179 AF at 2%, 5%, 10% and 20% (*v*/*v*) in PDA medium inhibited *A. alternata* mycelial growth by 88.08%, 74.04%, 61.97% and 34.62%, respectively (Table 2), confirming the ability of SZF-179 to secrete antifungal metabolites. 

### 2.5. Effect of SZF-179 on Mycelial Morphology by Ultra-Depth of Field Three-Dimensional Microscope

**Figure 5:**
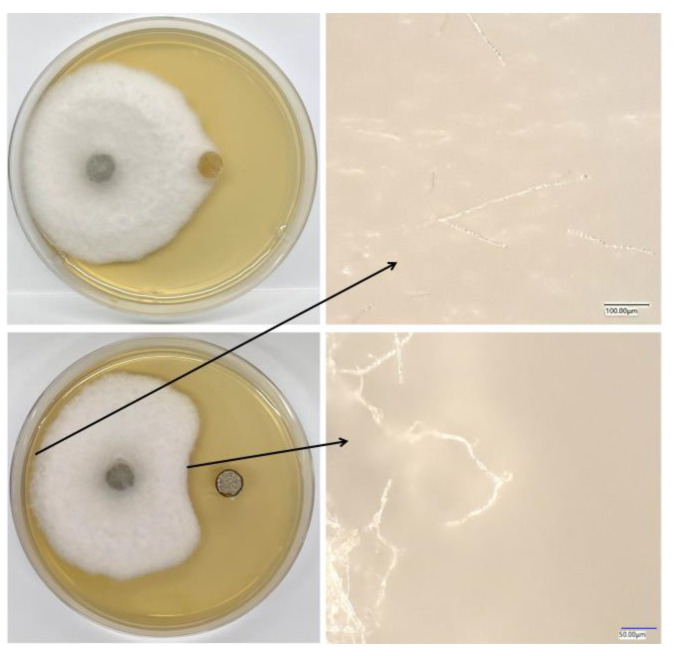
The mycelial morphology of *A. alternata* co-cultured with SZF-179. The long arrow represents the mycelial morphology of *A. alternata* far away from SZF-179, the short arrow represents the mycelial morphology of *A. alternata* co-cultured with SZF-179.

The inhibition of fungal pathogens by SZF-179 prompted us to examine its effect on the mycelial morphology of *A. alternata*. The ultra-depth of field three-dimensional microscope VHX-7000 studies showed that *A. alternata* mycelium near SZF-179 plug exhibited severe morphological abnormalities. In contrast, *A. alternata* mycelium located on the other side of the PDA plate did not show any irregularities (Figure 5), suggesting SZF-179 could exert antifungal effects by inhibiting the mycelial growth of *A. alternata.*

### 2.6. Identification of the Antifungal Polyene Compounds Containning Glycosyl Fragments

**Figure 6:**
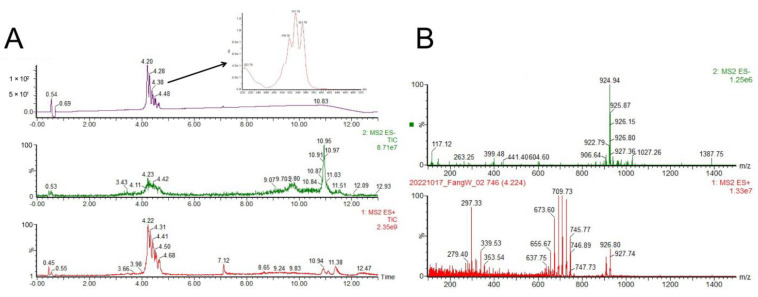
UPLC−MS analysis of the secondary metabolites from the strain SZF-179. (**A**) Liquid chromatography−mass spectrometry analysis of secondary metabolites from SZF-179 fermentation broth and typical compound UV spectrum (T 4.20 min). (**B**) A graph showing the data of LC−MS and identified compound pentene macrolide as a part of fraction glycosyl unit (loss a 180 fragment in ESI+). Typical compound mass spectrum (T 4.20 min).

To identify the antifungal compounds of SZF-179, ultra-high-performance liquid chromatography/tandem mass spectrometry (UPLC-MS) was applied to analysis the secondary metabolites. The main metabolites of SZF-179 eluted for 4–5 min (Figure 6A) had the same UV spectrum as a class of pentene macrolide, which have characteristic UV absorption spectra at 318 nm, 334 nm and 352 nm. Combined with its mass spectrum (Figure 6B), we supposed this compound contains glycosyl unit (loss a 180 fragments in ESI+). Due to the inherent instability and interconversion, the pure compounds could not be obtained, so chemical structure of these compounds was not identified in this study.

### 2.7. Thermal and pH Stability of SZF-179 Fermentation Broth

**Figure 7:**
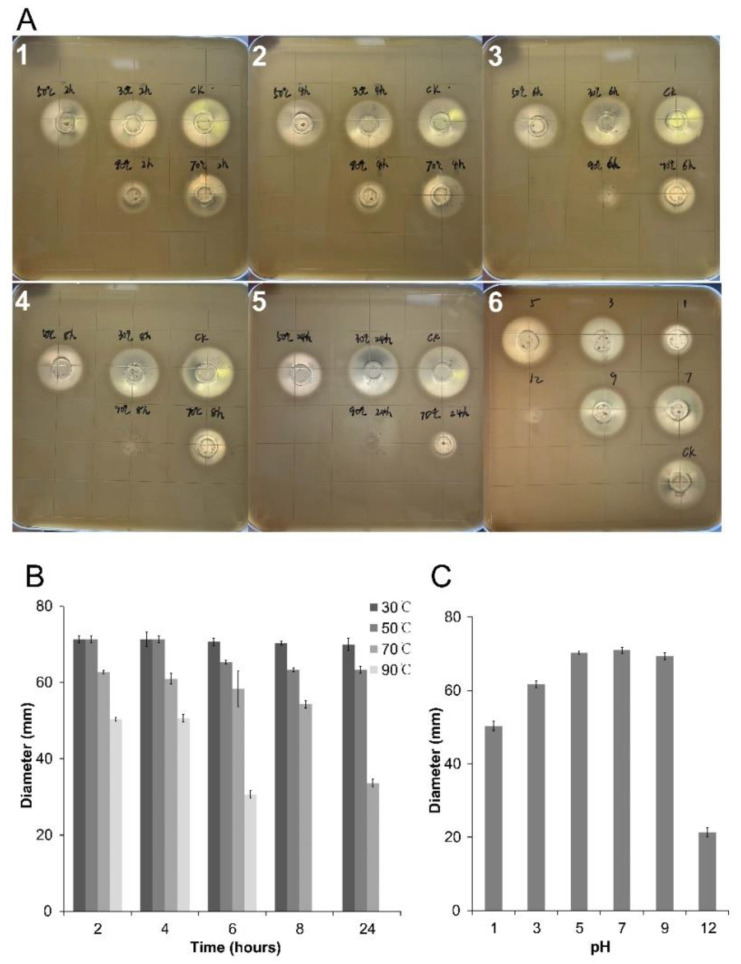
Antifungal activity of SZF-179 fermentation broth against *S. cerevisiae*. (**A**) **1**–**5**: thermo-stability test of SZF-179 fermentation broth: **6**: pH stability test of SZF-179 fermentation broth. (**B**,**C**) diameter of inhibition zones against *S. cerevisiae* mycelial growth at different temperature and pH. Each treatment mean value represents the average of 3 repetitions. The standard error is expressed in bars.

UPLC-MS results showed that the active substance in SZF-179 against *A. alternata* was a class of polyene; however, polyene compounds are not very stable and difficult to purify [24]; so, using *Saccharomyces cerevisiae* as target, we tested the stability of SZF-179 fermentation broth under different temperatures and pHs. Temperature sensitivity experiment results showed that, when the temperature was below 50 °C, the active substance was relatively stable, and the size of the antifungal zone was the same as that in the control. When the temperature was at 90 °C, the antifungal activity completely disappeared and there was no antifungal zone on the plates (Figure 7A). In addition, the pH sensitivity experiment results showed that the active substances were stable at pH 3–9 (Figure 7A). Compared with the control, the antifungal zone decreased by 80% at pH 1. When the pH increased to 12, it completely lost activity and no antifungal zone appeared. Collectively, although the active substances produced by SZF-179 lost their antifungal effect under high temperatures (90 °C) and in strong acids and bases, the antifungal activity remained very high under normal temperature and pH, which can continuously exert its antifungal effect in the environment and facilitate its’ commercialization.

### 2.8. Biocontrol Effects of SZF-179 on Pear Leaves 

**Figure 8:**
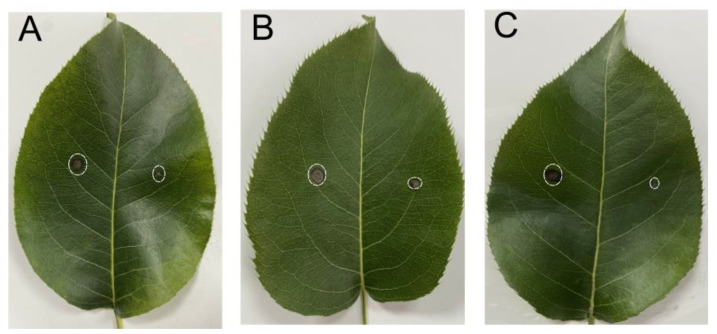
Efficacy of SZF-179 to control *A. alternata* on pear leaves. Right-halves of leaves were pre-treated with 1 × 10^6^ cells/mL (**A**), 1 × 10^7^ cells/mL (**B**) and 1 × 10^8^ cells/mL (**C**) SZF-179 fermentation broth. Left-halves of leaves were pre-treated with water.

SZF-179 showed strong activity against *A. alternata* through inhibiting its mycelial growth, so we conducted a protective experiment to study the biocontrol efficiency of SZF-179 against *A. alternata*. ‘Wonwhang’ pear leaves with similar morphologies and growth statuses were selected as experimental subjects. As shown in Figure 8, after inoculation for 3 days, the diameters of the necrotic lesions on the right-half of leaves pre-treated with fermentation broth of SZF-179 was reduced by 22.15%, 34.97%, and 42.25% when applying 1 × 10^6^, 1 × 10^7^ and 1 × 10^8^ SZF-179 cells/mL, respectively, compared to the negative control group (Table 3). Therefore, 1 × 10^8^ cells/mL bacterial concentration of SZF-179 was selected and used for further study. 

### 2.9. Field Control Effects of SZF-179 on A. alternata 

To confirm the biocontrol effect of SZF-179 on *A. alternata* in a field environment, we conducted a field experiment using SZF-179 fermentation broth on pear trees. After being sprayed three times with a foliar spray made of SZF-179 fermentation broth, the pear black spot disease index of treatment (1.55 and 0.79 on ‘Hosui’ and ‘Wonwhang’ pear trees, respectively) were significantly lower than that of water control (2.31 and 1.09 on ‘Hosui’ and ‘Wonwhang’ pear trees, respectively), and had no significant difference with that of 10% difenoconazole 2000× diluent treatment (1.30 and 0.70 on ‘Hosui’ and ‘Wonwhang’ pear trees, respectively) (Table 4).

## 3. Discussion

The pear (*Pyrus* spp.) is an economically important fruit cultivated worldwide. Pear black spot disease, caused by *A. alternata*, is a deleterious disease that occurs during the entire growing period, adversely affecting the sustainable development of the pear industry [25]. Biocontrol agents have been widely studied as a promising sustainable alternative and had been proven to have application value to control plant diseases [26]. *Bacillus siamensis* LZ88 exhibited a significant antifungal activity with an inhibition rate of 81.96% for the control of tobacco brown spot disease [27]. The postharvest treatment of strawberry fruits with *Meyerozyma guilliermondii* SQUCC-33 significantly reduced the fruit rot lesion size by 67.5% caused by *A. alternata* [28]. In addition, *Streptomyces* is one of the most speciose genera of *actinomycete* and has been also broadly used in the control of *Alternaria*-caused diseases. V C Verma et al. reported that three *Streptomyces* strains, AzR-051, AzR-049 and AzR-010, significantly antagonized the growth of *A. alternata*, the agent of tomato early blight disease [29]. Consistently, *S. odonnellii* SZF-179 was herein found to stronglyinhibite the growth of *A. alternata* in vitro and in vivo. As such, *S. odonnellii* SZF-179 offers a potential value as a novel biocontrol agent against pear black spot disease. 

*S. odonnellii* SZF-179 was confirmed to secrete antimicrobial compounds due to the culture filtrate and also showed a significant antifungal activity. In previous research, polyene macrolide compounds generally exhibited significant antimicrobial activity. Nystatin produced by *Streptomyces noursei* ATCC 11455 was reported as an important antifungal agent [30]. Heptaene macrolide antibiotic from *Streptomyces* sp. FR-008 has been identified to possessantifungal activity [31]. In our investigation, the main antifungal compounds in SZF-179 were identified as polyene-containing glycosyl fragments by UPLC-MS. In the following stability experiment, the SZF-179 AF also exhibited the same antifungal activity as the control, so we supposed that the polyene secreted by SZF-179 played the antifungal role. However, the pure polyene compounds could not be obtained, owing to the inherent instability and interconversion. Later, we will sequence the genome of SZF-179 and validate this hypothesis by knocking out the key genes that determine the production of polyene compounds.

The antifungal mechanism in substances from *actinobacteria* is mostly related to mycelium morphological and cell structure alterations [32,33,34]. Getha and Vikineswary observed severe mycelium distortions in *Fusarium oxysporum* caused by *S. violaceusniger* G10 extracellular metabolites [35]. Rajesh K. Manhas obtained a similar conclusion when investigating biocontrol potential of *Streptomyces hydrogenans* Strain DH16 toward *Alternaria brassicicola* to control damping off and black leaf spot in *Raphanus sativus* [36]. Likewise, in this study, microscopic observations of fungal mycelia from the margins of the inhibition zones (resulted from plug of strain SZF-179) revealed severe mycelial deformation. However, the integrity of the mycelium was not damaged. It is speculated that its antifungal mechanism is achieved by interfering with the normal physiological active components within the bacterial cells, resulting in the inability of the bacterial cells to carry out normal life activities and achieving antifungal effects. This deeper mechanism of action needs further exploration.

In recent years, the application of crude metabolites produced by *Streptomyces* spp. is also gaining attention in plant protection, which may be a supplement to chemical pesticides [37,38]. Therefore, in this study, the biocontrol potential of fermentation broth from *S. odonnellii* SZF-179 against *A. alternata* was evaluated. Pre-treatment with *S. odonnellii* SZF-179 fermentation broth led to a statistically significant (*p* ≤ 0.05) reduction in the diameters of the necrotic lesions on pear leaves. As such, the *S. odonnellii* SZF-179 fermentation broth achieved a similar effect to chemical difenoconazole in the control of black spot disease on ‘Hosui’ and ‘Wonwhang’ pears in a field application, indicating that the SZF-179 strain has a good application prospect and development potential in the control of pear black spot disease. 

Collectively, this is the first report on the biocontrol ability of *S. odonnellii* for the management of pear disease. SZF-179 is expected to be developed into a safe and broad-spectrum biocontrol agent. 

## 4. Materials and Methods

### 4.1. Isolation and Identification of Fungal Pathogens

In August 2021, in a pear orchard in Jiangxia district, Wuhan, China (114°8′37″ E, 30°17′44″ N, WGS84), pear leaves with typical brown-black circular spots symptoms were collected and excised into pieces (5 × 5 mm) at the junction of diseased and healthy tissues, disinfected in 75% ethanol for 30 s, rinsed in sterilized water two times, air dried for 30 min and placed on Potato Dextrose Agar (PDA) in Petri dishes (9 cm). After 5 days at 25 °C in darkness, mycelia were transferred to fresh PDA and purified via single-spore isolation. 

Genomic DNA of mycelia from purely isolated fungi was extracted using the FastPure bacteria DNA Isolation Kit (Vazyme Biotech Co., Ltd., Nanjing, China). Amplification of the internal transcribed spacer (ITS) was performed using the primer pair ITS1 (5′-TCCGTAGGTGAACCTGCGG-3′) and ITS4 (5′-TCCTCCGCTTATTGATATGC-3′). PCR amplification and Sanger sequencing were performed as in previously reported methods [39].

Healthy leaves and fruits of ‘Wonwhang’ pear were collected from the field. After being disinfected in 75% ethanol for 30 s and rinsed twice in sterilized water, healthy leaves and fruits were pricked gently with a sterilized insect needle 6 times to produce wounds. A 5 mm diameter colony of *A. alternata* identified was placed on the surface of wounded leaves and fruits. At the same time, blank PDA plugs, in the absence of *A. alternata*, served as a control. The inoculated leaves and fruits were maintained in a growth chamber at 25 °C for 3 days, after which brown necrotic lesions appeared. The pathogen was re-isolated and re-identified from the inoculated leaves and fruits, ensuring that the same pathogen could be identified and the same symptoms could be produced as in naturally infected pears.

### 4.2. Isolation and Screening of Antagonistic Bacteria 

The rhizosphere soil of pear trees with good appearance and absence of disease symptoms were randomly collected in the same orchard in Section 4.1, and the antagonistic bacteria were isolated as previously described [40]. In brief, the soil samples were diluted with sterile water and spread on nutrient agar (NA) culture medium (soybean powder 25 g/L, mannitol 25 g/L, agar 20 g/L) with the supplement of cycloheximide and nalidixic acid. After 3 days of cultivation at 28 °C, single colonies were chosen for purification. The purified bacteria were kept on NA medium for the following experiment. The inhibitory ability of bacteria purified against *A. alternata* was evaluated by the dual culture method, where the pathogen agar plug was placed on one side of PDA plate and the plug of antagonist was placed on the other side of the same plate. The PDA medium inoculated only with fungal pathogen served as a control.

### 4.3. Identification of Antagonistic SZF-179

After being cultivated on NA medium at 28 °C for 48 h, morphological characterization of SZF-179 was carried out using a Scanning Electron Microscope (SEM) (JSM-6390LV, Tokyo, Japan). Subsequently, the SZF-179 strain was identified by using PCR amplification of the 16S rRNA sequence with primer pair 27F (5′-AGAGTTTGATCCTGGCTCAG-3′) and 1492R (5′-GGTTACCTTGTTACGACTT-3′) [41]. PCR amplification and Sanger sequencing was conducted as in Section 4.1.

The 16S rRNA gene sequence was searched against the NCBI nucleotide database using the BLAST program and a neighbor-joining phylogenetic tree was constructed in MEGA version 7.0 software [42].

### 4.4. Inhibition Spectrum of the Antagonistic SZF-179

Four fungal and three bacterial pathogens were used to determine the inhibition spectrum of SZF-179 (Table 1). All pathogens were stored at Hubei Biopesticide Engineering Research Centre. The ability of SZF-179 to inhibit fungal pathogens was evaluated as in previously described methods [33], with a few modifications. Briefly, all fungal pathogens were activated and prepared in 5 mm mycelial disks using sterilized pipette tips and inoculated in the center of a 900 mm PDA plate. Later, 4 blocks of SZF-179 activated on ISP2 plates for 5 days were simultaneously placed in the same plate in four directions, 20 mm away from the disks with tested pathogens, and cultured at 28 °C for 5 days. Treatments of plugs of the fungal pathogen and blank NA medium placed in the same plate were used as control. Inhibitory rate was calculated according to the following formula: 100% × [(control colony diameter − treated colony diameter)/control colony diameter]. 

The ability of SZF-179 to inhibit bacterial pathogens was tested using the Oxford cup method [43]. Oxford cups were placed on the solid Luria–Bertani (LB) agar plates (containing 1.5% agar) containing 1‰ (*v*/*v*) bacteria cell culture (OD_600_ = 1.0) grown in LB medium. After cultured at 28 °C overnight, if a transparent inhibition zone appears around the Oxford cup, it is determined that SZF-179 has antibacterial activity. Three replicates were performed for each pathogen.

### 4.5. Effect of SZF-179 on Mycelium Morphology of A. alternata

SZF-179 and *A. alternata* were co-cultured on a PDA plate as described above in the dual culture method in Section 4.2 for 5 days, then mycelium near the SZF-179 plug and near the pathogen plug were separately chosen and visualized using the ultra-depth of field three-dimensional microscope (VHX-7000, Osaka, Japan).

### 4.6. Evaluation of Antifungal Activity of SZF-179 AF against A. alternata 

Five cultured blocks of SZF-179 from ISP2 plate were first placed into SFM medium (mannitol 10 g/L, glucose 10 g/L, soy peptone 10 g/L) in 250 mL Erlenmeyer flasks and cultured at 28 °C for 2 days with shaking (150 r/min). And then, at a ratio of 1:10 (*v*/*v*), the seed liquid was further transferred into ten 5 L Erlenmeyer flasks, each of which contained 2 L of the fermentation medium (potato starch 35 g/L, glucose 10 g/L, sesame meal 5 g/L, yeast extract 2.5 g/L, potassium dihydrogen phosphate 1.0 g/L, calcium carbonate 1.0 g/L), and cultured at 28 °C for 7 days on the rotary shaker (120 r/min). At last, the obtained liquid fermentation broth of SZF-179 was centrifuged at the 10,000 r/min for 10 min and the supernatant was filtrated through a 0.2 µm microporous membrane to get the AF.

The SZF-179 AF was diluted at 2%, 5%, 10% and 20% (*v*/*v*) in PDA medium. And then, a 5 mm diameter plug of *A. alternata* was inoculated in the center of Petri dishes containing amended medium. *A. alternata* plugs placed on non-amended PDA plates (sterile water added) were used as a control. All plates were cultured at 28 °C. Once *A. alternata* colonies on the control PDA plates had grown to cover the entire plate, the colony diameters on the amended PDA plates were measured and the inhibition rate of SZF-179 AF against *A. alternata* was calculated as described in Section 4.4. The experiment was repeated three separate times. 

### 4.7. Analysis of the Secondary Metabolites from SZF-179 by UPLC-MS

To identify the main antifungal compounds in SZF-179 supernatant, 10 mL of culture broth was extracted with 10 mL ethyl acetate. The ethyl acetate was evaporated and dissolved in 1 mL methanol. Crude extracts were filtered through 0.22 µm syringe filters and injected into a C18 reverse phase column (Waters Acquity UPLC BEH C18 1.7 μm, 2.1 × 100 mm, 0.45 mL/min) on Waters Acquity UPLC systerm. The mobile phases were H_2_O (0.2% acetic acid, A) and acetonitrile (ACN, 0.2% acetic acid, B). The linear elution gradient was: 0–0.2 min, 95%A; 0.2–4.2 min, 95%A–100%B; 4.2–5.2 min, 100%B; 5.2–5.70 min, 100%B–95%A; 5.70–7.00, 95%A for 10 min at a flow rate of 0.45 mL/min. 

### 4.8. PH and Thermal Stability of SZF-179 AF 

AFs from SZF-179 with varying pH gradients (1, 3, 5, 7, 9 and 12) were prepared and incubated at ambient temperature, followed by agar diffusion growth inhibition assay against *Saccharomyces cerevisiae*. *S. cerevisiae* was first activated in TSBY broth liquid medium (1.5 g/L tryptone, 0.5 g/L soya peptone, 0.5 g/L sodium chloride, 2.5 g/L yeast extract). Then, 45 mL autoclaved cool PDA medium (1% agar) premixed with 90 μL overnight culture of *S. cerevisiae* was poured into the square Petri dish (10 cm × 10 cm) to solidify. Oxford cups were placed on the solidified PDA medium plates, and 30 μL AF with different pH was then dripped into each cup. All plates were kept at 28 °C for 16–24 h to observe inhibition zones formation. The experiments were repeated in triplicates.

1 mL SZF-179 AF was added into 1.5 mL sterile Eppendorf tube and incubated at 30 °C, 50 °C, 70 °C, 90 °C for 2 h, 4 h, 8 h, 10 h and 24 h, respectively. Then, 100 μL AF was taken out after incubation for agar diffusion growth inhibition assay against *S. cerevisiae*. Solid medium premixed with *S. cerevisiae* was prepared as described above, and then 30 μL AF treated at different temperature was dripped into each well. All plates were kept at 28 °C for 16–24 h to observe inhibition zones. The experiments were repeated in triplicate.

### 4.9. Efficacy of SZF-179 for the Control of A. alternata on Pear Leaves

Healthy ‘Wonwhang’ pear leaves were chosen, sterilized and wounded following the conditions described in Section 4.1. Then the SZF-179 AF was smeared on the right-half of the leaves, and sterilized ddH_2_O smeared on the left-half of the leaves served as a control. Afterwards, the pear leaves were air-dried at room temperature (25–28 °C) for 1 h. Then, 5 mm diameter plugs of *A. alternata* were inoculated on the surface of wounded leaves, and the leaves were kept at 75% humidity and 28 °C for 3 days. The inhibition rate was calculated according to the diameter of the necrotic lesions. Experiments were repeated three times.

### 4.10. Field Assessment of the Biocontrol Efficacy of SZF-179 against Pear Black Spot Disease

A field trial was conducted in a pear orchard in Xiantao (113°21′14″ E, 30°23′86″ N, WGS84), Hubei province. The local soil is red loam, the average annual temperature is 14–18 °C, and the average annual rainfall is 800–1500 mm. Twelve-year-old pear trees of ‘Wonwhang’ and ‘Hosui’ (30 each) were taken as research plants. Plant row spacing of pear tree is 2 m × 4 m and tree shaped flat trellis with a height of 1.8–2 m. 

Three times foliar spray on the pear trees on 2 June, 22 June and 14 July 2022, were conducted as follows: treatment: SZF-179 (1 × 10^8^ cells/mL); negative control: water; positive control: 10% difenoconazole (2000× diluent). Each treatment was applied on 10 pear trees. The severity of pear black spot was investigated 10 days after the last spray. 50 leaves from each tree were randomly selected. The evaluation criteria for disease severity levels and calculation of disease indexes were performed according to the previous method [44].

### 4.11. Statistical Analysis

SPSS (Statistical Package, Version 20.0, Armonk, NY, USA) was adopted to conduct statistical analyses. All obtained results were calculated as the mean value and SD. The acquired data were processed using one-way analysis of variation (ANOVA), and the statistical significance was determined at *p* < 0.05.

## 5. Conclusions

This study concentrates on exploring the potential of new biocontrol strategies on pear black spot disease. A biocontrol *Streptomyces* with broad-spectrum antifungal activity was isolated from the rhizosphere soil around the pear trees. It was identified and named as *S. odonnellii* SZF-179. The strain exhibited a strong antifungal activity against *A. alternata,* through main active compounds released were polyene-containing glycosyl fragments, which severely affected the mycelial growth and lead to mycelium deformation and agglutination. The above results indicated that SZF-179 could be a potential biocontrol agent against *A. alternata*. Moreover, the protective experiment showed that the diameters of the necrotic lesions on pear leaves could be significantly reduced, when treated with AF of SZF-179. Furthermore, SZF-179 has a similar control efficiency to chemical difenoconazole in the control of black spot disease in the field, confirming that SZF-179 has been proven to be an efficient antifungal agent in practical applications. In sum, a new *Streptomyces* strain, *S. odonnellii* SZF-179, which produces bioactive compounds, is a potential antifungal agent for the treatment various plant diseases in ecofriendly agriculture.

## Figures and Tables

**Table 1:** Inhibition rate of SZF-179 against four fungal and three bacterial pathogens.

Pathogens	Relative Inhibition Rate (%)
*Colletrichum gloeosporioides*	36.62 ± 0.80 c
*Fusarium oxysporum* f. sp. *lycopersici*	42.62 ± 0.80 b
*Pythium aphanidermatum*	50.97 ± 0.67 a
*Alternaria alternata*	55.04 ± 0.31 a
*Staphylococcus aureus*	—
*Micrococcus luteus*	—
*Escherichia coli* DH10B	—

Note: Different letters on the column indicate the existence of significant differences between the relative inhibition rates, which has been tested at *p* < 0.05. “—” indicates no inhibitory effect.

**Table 2:** Inhibition rate of SZF-179 AF against *A. alternata* mycelial growth.

Treatment	Diameter in mm ^a^	Relative Inhibition Rate (%) ^b^
Control ^c^	77.62 ± 0.40 a	—
2%AF	50.75 ± 0.27 b	34.62 ± 0.80 d
5%AF	29.52 ± 0.18 c	61.97 ± 0.67 c
10%AF	20.15 ± 0.06 c	74.04 ± 0.31 b
20%AF	9.25 ± 0.47 d	88.08 ± 0.34 a

^a^ Mycelial diameter (each treatment value represents the average of 10 replicates) ± standard deviation (SD). ^b^ Differences between means were considered significant (*p* ≤ 0.05). Inhibition assays were considered for different statistical groups. Different letters in the same column indicate that the variables are significantly different. ^c^ The control experiment was carried out in the absence of AF supernatant. “—” indicates no inhibitory effect.

**Table 3:** Inhibition rate of SZF-179 to control *A. alternata* on pear leaves.

Treatment (cells/mL)	Lesion Diameter (cm) ^a^	Inhibition Rate (%) ^b^
Control ^c^	0.632 ± 0.198 a	——
1 × 10^6^	0.492 ± 0.198 b	22.15 ± 0.98 c
1 × 10^7^	0.411 ± 0.076 b	34.97 ± 0.76 b
1 × 10^8^	0.365 ± 0.078 c	42.25 ± 1.24 a

^a^ Lesion diameter (each treatment value represents the average of 10 replicates) ± SD. ^b^ Differences between means were considered significant (*p* < 0.05). Preventive assays were considered for different statistical groups. Different letters in the same column indicate that the variables are significantly different. ^c^ The control experiment was carried out with water.

**Table 4:** Control effects of different treatments on pear black spot in field.

Cultivar	Treatment	Disease Index
Hosui	SZF-179 fermentation broth	1.55 b
	10% difenoconazole 2000× diluent	1.30 b
	water	2.31 a
Wonwhang	SZF-179 fermentation broth	0.79 b
	10% difenoconazole 2000× diluent	0.70 b
	water	1.09 a

Note: Different letters indicate significant differences (*p* < 0.05).

## Data Availability

The data analyzed in this study are included within the paper.
